# Rhinologists: Who are we?

**DOI:** 10.1016/S1808-8694(15)30482-1

**Published:** 2015-10-19

**Authors:** Olavo Mion, Nilvano Andrade

**Affiliations:** Secretary – Brazilian Academy of Rhinology, Professor of Otorhinolaryngology – University of São Paulo Medical School, PhD Assistant – Allergy Studies Group – Otorhinolaryngology Ward – University of São Paulo Medical School Hospital; Chairman – Brazilian Academy of Rhinology, PhD in Otorhinolaryngology – University of São Paulo Medical School, Adjunct Professor – School of Medicine and Public Health of Bahia

In recent years rhinology has become an area of great interest for otorhinolaryngology, and its topics have enjoyed great popularity in our specialty. There are many reasons for such fact: most of the attraction felt by otolaryngologists from Brazil and abroad for rhinology is due to the fast technological development which improved diagnostic methods and consequently enhanced the diagnosis of nose and paranasal sinuses diseases. There was a broadening in the types and the many possibilities for clinical and, especially, surgical treatment. The areas which had the most development in recent years have been training – with an increase in the number of techniques, new equipment and indications for endoscopic nasal surgery, and the very possibility of operating with the help of a navigator. We must stress that this increase in the number of treatment possibilities and the new techniques and equipment available must be tested and judged by physicians and specialists, surgeons and renowned researchers in the field. Only then we will reach a conclusion and a decision on the true best techniques and the best devices for training, teaching and doing research in Brazil and abroad.

Another fact which stimulates interest in rhinology is the high prevalence and incidence of nose and paranasal sinuses diseases. Jointly, we can not refrain from stressing that allergic and non-allergic inflammatory disorders in the specific area of rhinology have been on the rise. One of the things which is still very much unknown to us, or partially unknown, is the very reason for this increase in the incidence of allergic disorders. What we still do not know, at least in Brazil, is how much it increased and if they continue to increase, among other doubts in this specialty. These facts still remain obscure and require intense research, especially epidemiological studies. One of the promising areas and which still has much to develop is the field of olfaction and related areas, in which research is very difficult to lead and with high levels of difficulties in methodology, and also because of the lack of proper equipment for proper objective measures, for instance.

Mentioning olfaction as a promising research area, we notice that one area in Brazil that still requires stimulus is scientific research and publication, and rhinology is not different.

**Figure 1:**
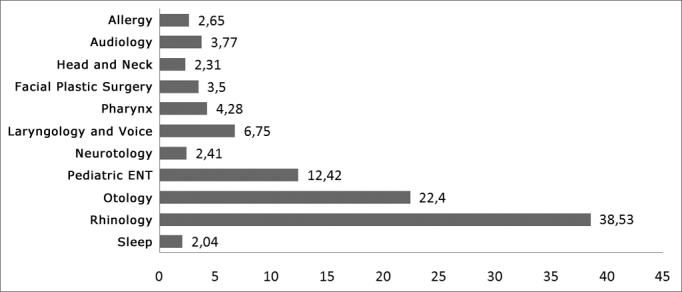
Working field (2007 census from the ABORLCCF)

In the year of 2007 the Brazilian Association of Otorhinolaryngology held its second census, and according to this survey carried out with all its members at the time – 6,857 otorhinolaryngologists which responded the questionnaire, about 38.63% of the otorhinolaryngologists worked with rhinology and about 2.65% worked with allergy, making up about 41.28% of all the otorhinolaryngologists in Brazil, a grand majority of the specialists in Brazil, in comparison with the other specialties (see figure 1).

One could expect that all this clinical and surgical interest would reflect on a greater scientific production, if not in basic disciplines, but above all in clinical research, maybe epidemiological or even research approaching surgical treatment.

As far as the Brazilian scientific production is concerned, we can see a somewhat different picture. In the Brazilian Journal of Otorhinolaryngology, if we use the search engine with some words associated with rhinology we will find some very interesting results. We see that in the past 3 years (2007, 2008, 2009) the words rhinology, nose, rhinitis, paranasal sinuses were found 182 times in national publications. In the year of 2007, there were 85 hits, in 2008 there were 69 and in 2009, up until March/April, there were 28 hits by the search engine on the journal website (www.rborl.org.br).

**Figure 2:**
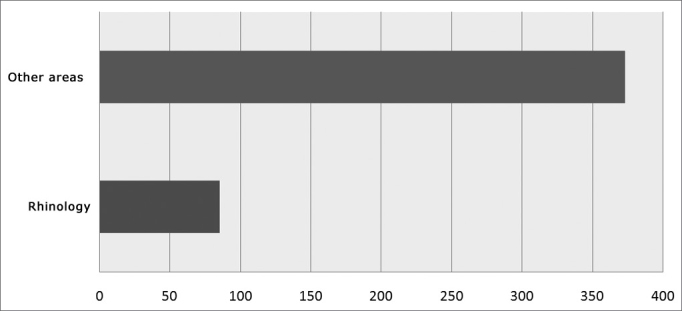
Number of papers published in the Brazilian Journal of Otorhinolaryngology

In total, along these 3 years there were 85 papers published in the field of rhinology, 35 in 2007, 36 in 2008 and 14 in 2009. In the year of 2007, a total of 146 papers were published in the Brazilian Journal of Otorhinolaryngology, in the year of 2008, 170 papers and, in 2009 up until March/April, there were 57 papers, making up a total of 373 papers. Therefore, the number of publications in rhinology was 22.7% in the recent 3 years (see fig. 2) This superficial analysis shows that the national publication of papers in this field does not reflect the interest of Brazilian otorhinolaryngolgists in general. The number of publications is lower than the work of physicians in the field of rhinology, which is more than 40% – in other words, the papers published in this field have a lower importance in relation to the other specialties.

Therefore we may conclude that it is necessary to have greater stimuli to research in the area of rhinology, without losing sight of the clinic, surgery and also the campaigns used to educate the population, such as the “Breath through your nose and live better”. This extremely important initiative must be better divulged and enjoy the most attention from rhinologists, since it aims at educating the population in general about the role of rhinologists in treating nasal obstruction.

